# The perception of quality of care and respect of human rights in mental healthcare facilities among users during and after the pandemic in Sardinia, Italy

**DOI:** 10.3389/fpubh.2026.1773601

**Published:** 2026-05-21

**Authors:** Elisa Cantone, Vanessa Barrui, Michela Atzeni, Giulia Cossu, Viviana Forte, Federico Contu, Stefano Lorrai, Alessandra Perra, Elisa Pintus, Haidy Rocio Oviedo Cordoba, Giulio Oppes, Felice Curcio, Rober Romero Ramirez, Shellsyn Giraldo Jaramillo, Massimo Tusconi, Cesar Ivan Aviles Gonzalez

**Affiliations:** 1 Doctoral School in Capacity Building for Global Health, Department of Medical Sciences and Public Health, University of Cagliari, Cagliari, Italy; 2 Department of Mental Health and Addiction, ASL Ogliastra, Lanusei, Italy; 3 Cagliari University Hospital, Cagliari, Italy; 4 Department of Medical Sciences and Public Health, University of Cagliari, Cagliari, Italy; 5 University of Magdalena, Santa Marta, Colombia; 6 Faculty of Medicine and Surgery, University of Sassari (UNISS), Sassari, Italy; 7 Universidad Popular del Cesar, Valledupar, Colombia; 8 Department of Medicine and Surgery, University of Enna “Kore”, Enna, Italy

**Keywords:** community mental health services, COVID-19 pandemic, health care resources, health system resilience, human rights, Italy, mental health service users, Sardinia

## Abstract

**Background:**

The coronavirus disease 2019 (COVID-19) pandemic has strongly challenged health systems, but Italian community mental health services, rooted in the Basaglia reform and the World Health Organization (WHO) QualityRights framework, may have preserved a high perception of care quality and respect for human rights. This study compared users’ perceptions of quality of care, human rights, and resource adequacy in public mental healthcare facilities during and after the pandemic in Sardinia, Italy.

**Methods:**

A cross-sectional survey was conducted in 2025 among users attending three community mental health services (Nuoro and Sanluri Local Health Networks) and one university hospital facility (Cagliari). Data were compared with a previous survey carried out in the same network during the COVID-19 pandemic (2021). Users completed a brief sociodemographic form and the “Well-Being at Work and Respect for Human Rights Questionnaire” (WWRR, user version), inspired by the United Nations (UN) Convention on the Rights of Persons with Disabilities and WHO QualityRights. Group differences were analyzed using chi-square tests and one-way analysis of variance (ANOVA).

**Results:**

The final 2025 sample included 118 users, compared with 200 users in 2021. Mean satisfaction scores for overall care and organizational aspects (WWRR items 1–3) remained very high in both surveys (>5/6) without significant differences. Perceived respect for users’ rights (item 4) and staff rights (item 5) significantly increased post-pandemic (5.26 ± 1.10 vs. 5.51 ± 0.98, *p* = 0.042; and 4.89 ± 1.22 vs. 5.35 ± 1.04, *p* = 0.001, respectively). Perception of resource adequacy (item 6, reverse-coded) also improved (2.68 ± 1.10 vs. 2.06 ± 0.98, *p* < 0.001), although it remained the most critical domain. Item 7 indicated an evolving demand for a more diversified workforce, with psychologists being the most frequently requested professionals, and an emerging perceived need to increase multiple professionals across multiple categories and administrative/management staff.

**Conclusion:**

Users of Sardinian community mental health services reported persistently high satisfaction and an increased perception of respect for human rights after the COVID-19 pandemic, despite ongoing concerns about limited resources. These findings highlight the resilience of community-based mental healthcare and underscore the need for structural investment and the strengthening of the multidisciplinary workforce strengthening to sustainably align service provision with international human rights standards.

## 1 Introduction

The coronavirus disease 2019 (COVID-19) pandemic has had a major impact on healthcare services worldwide (1–3). Among users of care services, even those not directly affected by COVID-19, the need to visit healthcare facilities increased the fear of becoming infected (4–6). Accessibility to services was limited due to distancing measures, and this reduced accessibility was further exacerbated by the fact that many resources, including human resources, were redirected to cope with the pandemic (7, 8). Healthcare staff lived in constant fear of infecting themselves and their loved ones, and they were often subjected to extremely demanding work shifts and suffering by stress and mood and anxiety disorders (9–12). In Italy, mental health services came from a long-standing tradition of community-based care (13–15). Despite the limited resources found in the already documented in previous years in mental health services (16–19) the emergency of pandemics was often addressed with good adaptability and, where possible, through the adoption of remote care tools (20–22). This may have surprisingly led to a more optimistic perception among users and staff of mental health centers regarding satisfaction with care and the perceived respect for the rights of both users and professionals, compared to users and staff in non-mental healthcare services (23, 24). The importance of individuals’ perspectives in seeking care has also been emphasized by the United Nations (UN) Convention on the Rights of Persons with Disabilities (CRPD) (25). The World Health Organization’s QualityRights Project has introduced the principles of the CRPD into the field of mental health (26–28) by emphasizing dignity, recovery, autonomy, and participation of people with mental health problem on decisions on policies and mental health systems (18, 29, 30). The research project underlying the whole research on satisfaction by care and perception on rights in mental health services to which this article belongs was inspired by the QualityRights Project. It aimed to assess, across different international contexts and historical periods, the perspectives on care, job satisfaction, and perceived respect for the rights of users, informal caregivers/relatives, and healthcare workers in both mental health and non-mental healthcare services (31–36). The present study examines the perceptions of users of a public mental health service network in an Italian region during the COVID-19 period, based on previously published data (24), and compares them with results obtained from a subsequent sample collected in the same services after the COVID-19 pandemic.

## 2 Materials and methods

### 2.1 Study design

*Study sample.* A sample of users was voluntarily recruited from three community mental health services in the Local Health Networks (“Aziende Sanitarie Locali”) of Nuoro and Sanluri, in the Sardinian region, and from users of a mental healthcare facility of the University Hospital of Cagliari. The study tools were administered after authorization of the regional ethics committee during the same week in 2025, with the cooperation and consent of the health workers and head professionals of each collaborating unit. The Institutional Ethical Committee of the Regione Autonoma della Sardegna has approved the study protocol, under protocol number 25,569, dated 18 September 2025. The study was conducted over a defined one-week period in 2025 (October 7–14) to ensure consistency in recruitment conditions. Although this period does not correspond to the same calendar week as the 2021 survey, the same recruitment strategy and service settings were used to enhance comparability between the two samples. All users attending services for care (excluding first-contact services) that week were asked to participate. The exclusion criteria were admission to the facility for the first visit without previous contact with the healthcare unit, or having a severe mental health condition that makes it difficult to participate. According to these criteria, three users of those already in contact with the facility were excluded; none of the others refused to participate. Severe cognitive impairment was assessed based on clinical judgment by the treating mental health professionals. In routine clinical practice, clinicians evaluated each potential participant’s ability to understand the study procedures and to provide informed and reliable responses to the questionnaire. No standardized cognitive assessment instrument was applied for this purpose.

### 2.2 Study instrument

Probands were asked to complete the following:

A simple tool for collecting basic information about demographic data (age, gender, and educational level). The same tool was already utilized in the cited previous survey (23).

The “Well-Being at work and respect for human rights questionnaire” (WWRR) (32, 36, 37) was adopted in the user version. The WWRR was conceived in accordance with the World Health Organization’s QualityRights project (26, 29) and the UN Convention on the Rights of Persons with Disabilities (25). An accurate description of the WWRR is available in previously published articles (37, 38). The different versions of WWRR, measured among health workers, users, and informal caregivers, assessed the organizational climate in the health services delivering care, the satisfaction with the care delivered (or, for staff, the satisfaction at work), and the perceived respect for rights in the facility. The items from 1 to 5 are coded on a 1–6 graduated scale, with 1 indicating “Not satisfied at all” and means “Completely satisfied.” Item 6 inquires about the perception of the adequacy of resources in the mental healthcare facility; this item is coded on a graduated scale from 1 to 5, in which 1 (in an inverse way) indicates “Completely satisfied” and 5 “Not satisfied at all.” Item 7 inquired about the perception of which kind of professional workers the interviewed thinks are lacking in the mental healthcare facility.

### 2.3 Statistical analysis

Statistical analysis was carried out by comparing nominal data using chi-square statistics with Yates’s correction when needed, and numerical discrete data were compared using one-way analysis of variance (ANOVA) statistics with Bonferroni correction. Although Likert-scale data are ordinal by definition, they were treated as approximately continuous for parametric analyses. This approach is supported by methodological literature indicating that parametric tests such as ANOVA are robust to violations of normality and can be appropriately applied to Likert-type data, particularly when sample sizes are moderate. Distributions are not markedly skewed (39, 40). In addition, results were interpreted cautiously, given the ordinal nature of the data. No standardization for sex was carried out because the two samples, due to the recruitment methods, are representative of the two different populations. Furthermore, the differences between the two samples in terms of age and sex are very slight.

### 2.4 Ethics statement

The Institutional Ethical Committee of the Regione Autonoma della Sardegna has approved the study protocol (protocol number: 25,569, dated 18 September 2025). The project was conducted in accordance with the Declaration of Helsinki (World Medical Association 2020). All probands signed the informed consent (which model was approved by the Ethical Committee). Clinical stability was assessed by the treating mental health professionals through routine care, based on clinical judgment. In particular, participants were considered clinically stable if they did not present acute psychopathological conditions (such as severe agitation, florid psychotic symptoms, or marked behavioral or cognitive disorganization) that could compromise their ability to understand and reliably complete the questionnaire. No standardized assessment tool or formal protocol was used for this evaluation. This study clearly stated that data would be collected anonymously and that each participant could withdraw from the study at any time. Participants’ answers or explanations could have been addressed to a single telephone number or to an email address dedicated to this purpose.

## 3 Results

Three users who were already in contact with the facility were excluded due to severe health conditions, according to the protocol. None of the others refused to participate. The final sample consisted of 118 users. Table 1 shows the characteristics and differences between the basic sociodemographic variables and the comparison with the previous sample (35). The two samples were balanced with respect to the frequency of males and those with less than 9 years of education. The sample recruited during the pandemic had a slightly lower proportion of people aged 49 or older (35.5% vs. 50%, *p* < 0.011).

**Table 1:** Characteristics of the samples.

Item	Users 2021 (200)	Users 2025 (118)	Statistics	*p*
	*N* (%)	*N* (%)		
Sex (men)	120 (61)	62 (52.5)	*χ*^2^ = 1.686	*p* = 0.194
Age > 49	71 (35.5)	59 (51)	*χ*^2^ = 6.456	*p* = 0.011
<9 years	107 (53.5)	64 (54.2)	*χ*^2^ = 0.016	*p* = 0.899

Table 2 compares the mean scores for responses to items 1–6 of the WWRR questionnaire across the two samples (2021–2025). No significant differences were found in the mean scores of the responses to items 1 (How satisfied are you with the services you receive?); 2 (How satisfied do you think the users of the service you work for are?); 3 (How satisfied are you with the organizational aspects of the services you receive?), where the mean response score was always higher than 5/6 in both samples. In item 4 (To what extent do you believe that the human rights of the people assisted in the service where you are cared are respected), a higher score is observed in 2025 with a statistically significant difference in 2021 (5.26 ± 1.10 in 2021 vs. 5.51 ± 0.98; *p* < 0.042); the same trend emerges in the mean scores of the responses to item 5 (To what extent do you believe that the human rights of the staff working in the services you are assisted by are respected?) (4.89 ± 1.22 in 2021 vs. 5.35 ± 1.04; *p* = 0.001) and to item 6 (how do you evaluate the current state of care of the services in which you are cared with reference to resources?), the scores for which are reverse coded, that is, with a higher score meaning a more pessimistic view (2.68 ± 1.10 in 2021 vs. 2.06 ± 0.98, *p* < 0.001). It should be noted, however, that in item 6, despite the large increase in 2025, the average responses are lower in both samples than in the other items, considering that if the scores are reported on a 1–6 scale (with maximum satisfaction = 6), as in the other items, scores are around the average (i.e., 3), slightly higher in 2025 and slightly lower in 2021.

**Table 2:** Answers to items 1–6 WWRR in Mental Health Services users during COVID-19 and in post-COVID-19.

Research sample	Users 2021 (200)	Users 2025 (118)	2021 vs. 2025 ANOVA 1,316 df	*p*
WWFF 1 – How satisfied are you with the services in which you are cared?	5.14 ± 1.17	5.27 ± 1.04	*F* = 0.9 93	*p* = 0.320
WWFF 2 – How much you believe that the users of the service in which you work are satisfied?	5.26 ± 0.99	5.35 ± 0.90	*F* = 0.6 55	*p* = 0.419
WWFF 3 – How satisfied are you with the organizational aspects of the services in which you are cared?	5.14 ± 1.10	5.16 ± 1.27	*F* = 0.0 22	*p* = 0.813
WWFF 4 – To what extent do you believe that the human rights of the people cared in the service in which you are cared are respected?	5.26 ± 1.10	5.51 ± 0.98	*F* = 4.5 0	*p* = 0.042
WWFF 5 – To what extent do you believe that the human rights of the staff working in the services you are cared by are respected?	4.89 ± 1.22	5.35 ± 1.04	*F* = 11.739	*p* = 0.001
WWFF 6 – How do you evaluate the current state of care of the services in which you are cared with reference to resources?	2.68 ± 1.10	2.06 ± 0.98	*F* = 25.526	*p* < 0.001

Table 3 compares the frequency of responses to item 7 of the WWRR regarding the types of professionals the respondent believes should be increased in the service where they receive care. The ranking of each response type is also indicated in both surveys. In 2021, the most frequent response was Occupational Therapist/Rehabilitation Technician, while its frequency decreased in 2025, where it moved to second place in the ranking; the difference in frequency was statistically significant (35% vs. 20.3%; *p* = 0.006). In 2025, the response “Psychologist” ranked first, although its frequency did not change between the two observations. The response “Physician” ranked third across two observations, with no statistically significant difference in response frequency. There were no significant differences in the frequency of responses related to “Nurse,” “Personal Care Professional,” “Social Worker,” “Staff Security,” and “None needs to be increased.” In 2025, the frequency of responses related to “Administrative/Staff Managing” increases 181 (from 0% in 2021 to 4.2% in 2025; *p* = 0.014) and in the response “All need to be incremented, or many kind 182 of workers need to be incremented” (from 0% in 2021 to 11.9% in 2025; *p* < 0.001).

**Table 3:** Answers to item 7 of WWRR in mental health services users during COVID-19 and in post-COVID-19.

Role	Users 2021 (*N* = 142) *N* (%) [rank]	Users 2025 (*N* = 118) *N* (%) [rank]	2021 vs. 2025 Chi-square 1 df	OR 2021 CI (95%)
Medical doctors	27 (13.5) [III]	18 (15.2) [III]	*χ*^2^ = 0.188	OR = 0.8
			*p* = 0.665	7 (0.4–1.6)
Nurses	15 (7.5) [IV]	11 (9.3) [V]	*χ*^2^ = 0.328	OR = 0.7
			*p* = 0.567	9 (0.3-1.8)
OSS professional for personal care	9 (4.5) [V]	8 (6.8) [VI]	*χ*^2*^ = 0.762	OR = 0.62
			*p* = 0.783	2 (0.2–1.5)
Psychologists	55 (27.5) [II]	37 (31.35) [I]	*χ*^2^ = 0.537 *p* = 0.464	OR = 0.8 3 (0.5–1.4)
Occupational therapists/educators/technicians of rehabilitation	70 (35) [I]	24 (20.3) [II]	*χ*^2^ = 7.661 *p* = 0.006	OR = 2.1 1 (1.3–3.6)
Social workers	15 (7.5) [IV]	8 (6.8) [VI]	*χ*^2^ = 0.057 *p* = 0.811	OR = 0.1 1 (0.5–2.7)
Administrative/staff managing	0	5 (4.2) [VIII]	*χ*^2*^ = 6.090 *p* = 0.014	OR = 0.0 (N.C.)^**^
Staff security	5 (2.5) [VI]	5 (4.2) [VIII]	*χ*^2*^ = 0.735 *p* = 0.391	OR = 0.6 (0.2–2.6)
None needs to be incremented	4 (2) [VII]	0 [X]	*χ*^2*^ = 1.051 *p* = 0.305	OR = Inf (NC)
All need to be incremented, or many kinds of workers need to be incremented	0	14 (11.9) [IV]	*χ*^2*^ = 22.983 *p* < 0.001	OR = 0 (NC)

^*^Yathes’ correction. ^**^NC, not calculable; OR, odds ratio; CI, confidence interval; OSS, social and health care worker (Operatore Socio-Sanitario [in Italian]).

## 4 Discussion

The findings of this study confirm the relatively high level of satisfaction and perceived respect for human rights among users of Italian mental health services, both during and after the COVID-19 pandemic. This is consistent with the longstanding tradition of community-based psychiatric care in Italy, rooted in the Basaglia reform, which has shaped a service model characterized by proximity to users, close ties with formal and informal support networks in the community, individualized care, and an emphasis on psychosocial rehabilitation (15, 41–46). These excellent results are being achieved against the backdrop of a pandemic and a general resource crisis for the Italian healthcare system (47, 48), with clearly limited resources for mental health in 2021, and amid a climate of widespread public disapproval of the healthcare system as a whole and towards healthcare providers themselves, who went from being considered “heroes” during the first phased of the pandemic to being blamed as responsible of the disaster in delivering care, with even acts of violence as highlighted in scientific literature and media (49–53). The stability of the mean scores on items 1–3 of the WWRR questionnaire, related to general satisfaction and organizational aspects, suggests that despite the pandemic, users maintained a positive perception of service quality of care. This may reflect the adaptability of mental health services in reorganizing care pathways, including the implementation of remote or hybrid care modalities as reported by previous research (21, 22, 54). The continuity of care, even under restrictive conditions, may have played a protective role in preserving user satisfaction. Notably, items 4 and 5 showed a significant increase in the post-pandemic sample, indicating an improved perception of respect for the rights of both users and staff. This result may suggest that the pandemic paradoxically stimulated greater awareness of the ethical, relational, and human rights dimensions of mental health services. As indicated by previous studies inspired by the WHO QualityRights initiative and the CRPD, the recognition of autonomy, dignity, participation, and recovery-oriented practices has been increasingly emphasized in the mental health field worldwide. The present findings may represent a local reflection of this broader cultural shift. Despite these positive trends, Item 6, concerning the adequacy of resources, remained the most critical area. Although the average score improved in 2025, it still indicates a persistent perception of insufficient resources, consistent with long-standing concerns about the underfunding of Italian mental health services. The perception of limited resources, despite high satisfaction in other domains, suggests that service quality may rely heavily on professional commitment and organizational resilience rather than structural adequacy. In contrast, the perception of users of mental health services is perfectly in line with what the literature (55–58), media (59–61), and economic reports (62, 63) show on cuts in healthcare spending in Italy, which also impact mental healthcare in greater proportion (14, 18, 64). The responses to item 7 highlight a perceived need for a more diversified and multidisciplinary workforce. While psychologists became the most requested professional figure in 2025, the reduced demands for occupational therapists and the emerging need for administrative and managerial staff point to evolving user expectations. The new presence of responses, such as many professionals need to be increased,” may reflect a broader awareness of systemic complexity and a perceived need for integrated interventions.

### 4.1 Limitations

Several limitations must be considered when interpreting the results: Sampling Method: The study relied on voluntary participation during a single week of recruitment, which may limit representativeness. However, refusal rates were low. Cross-sectional *Comparison*: The design allows comparison of two cohorts but does not enable longitudinal inference at the individual level. *No standardization for sex and age*: Although differences between the samples were limited, demographic variations could still play a role in some of the reported perceptions. However, the study aimed to capture the climate within a representative sample of service users. From this perspective, it’s not surprising that during the pandemic (although 2021 did not represent the peak of the first wave), the sample included fewer older adults, who, due to their greater risk, were primarily treated remotely and did not physically visit the facility. The responses are therefore representative of the service’s actual climate. *Self-report measures*: Data are based exclusively on questionnaire responses, which may be influenced by social desirability bias or contextual factors at the time of administration. *Regional focus*: The study was conducted in a single Italian region, limiting the generalizability to other contexts. Data collection was not conducted in the exact same calendar week across the two surveys, which may introduce minor temporal variability. Clinical stability was determined by clinical judgment without standardized assessment instruments, which may limit reproducibility and introduce subjectivity. The exclusion of individuals with severe cognitive impairment was based on clinical judgment rather than standardized assessment tools, which may introduce some degree of subjectivity in participant selection.

## 5 Conclusion

The study suggests that Italian community mental health services maintained a high level of satisfaction and respect for users’ rights both during and after the COVID-19 crisis. The post-pandemic increase in perceived respect for rights may indicate a positive cultural evolution, possibly influenced by international frameworks such as the CRPD and WHO QualityRights. However, the persistent perception of insufficient resources highlights the need for structural investment and workforce development.

These results underscore the resilience of community-based mental health services but also highlight the need to strengthen their sustainability. Future research should examine longitudinal trajectories, integrate qualitative approaches, and consider broader geographic contexts. Policymakers should prioritize resource allocation and multidisciplinary staffing strategies to align real-world service provision with the ethical principles increasingly recognized by users and international guidelines.

## Data Availability

The original contributions presented in the study are included in the article/supplementary material, further inquiries can be directed to the corresponding author.
